# Accuracy of third molar eruption for legal age estimation using the Gambier method in Lebanese subadults

**DOI:** 10.1007/s10266-025-01229-8

**Published:** 2025-10-23

**Authors:** Stefano De Luca, Siddharth Paladugu, Sudheer Babu Balla, Maria Moukarzel, Nikolaos Angelakopoulos

**Affiliations:** 1 Department of Organisms and Systems Biology, Faculty of Biology, University of Oviedo, Oviedo, Spain; 2 Government Dental College, Vijayawada, India; 3 Dentistry and Oral Health, La Trobe Rural Health School, Bendigo, VIC 3550 Australia; 4 Private Dental Office, Beirut, Lebanon; 5 Department of Orthodontics and Dentofacial Orthopedics, University of Bern, Bern, Switzerland

**Keywords:** Dental age estimation, Legal age, Third molar eruption, Gambier et al.’s scoring system, Lebanon

## Abstract

Assessing whether an individual has reached the legal age of 18 is a complex, multifactorial process that requires the application of reliable, standardized, and reproducible methods. Among the various approaches, the assessment of third molar eruption has recently emerged as a useful preliminary tool for estimating whether an individual has reached the age of majority. This study aims to evaluate the accuracy of the Gambier et al. scoring system for legal age estimation based on third molar eruption in a sample of Lebanese subadult individuals. A retrospective analysis was conducted on 537 orthopantomograms (OPGs), comprising 298 males and 239 females, aged between 15 and 24 years. An increase in mean chronological age was observed with the progression of third molar eruption stages (1–3) and phases (A–D) in both sexes. Only in limited cases has a strong relationship been found between phase D and the probability that an individual is 18 years of age or older. In this Lebanese sample, phase D, which corresponds to complete emergence in the occlusal plane, was not always associated with individuals being 18 years or older: the 11.9% of males and females in this phase is above the legal age threshold. This technique may serve only as a preliminary tool for estimating the probable age of alleged minors of Lebanese origin, particularly in the context of migrant populations and child marriage contexts. Its application is recommended in accordance with the minimum age principle, as minimum age thresholds have been established for each stage and phase of third molar eruption. This method, however, ought to be applied only in combination with other internationally validated dental age estimation methods, thereby safeguarding against potential ethical implications associated with legal age assessment.

## Introduction

Addressing the situation of undocumented migrant minors poses a critical challenge for medico-legal systems operating within the European Union (EU) [1]. From the experts' perspective, the central dilemma lies in balancing two fundamental priorities — minimizing the potential harm associated with medical age assessment procedures, while ensuring that these assessments remain as effective and reliable as possible, and provide a satisfactory level of legal certainty [2].

Several methods are currently available for age estimation in living individuals. However, the reliability and applicability of these methods vary considerably and must be critically assessed on a case-by-case basis, taking into account the specific legal, and contextual factors involved [3].

The Arbeitsgemeinschaft für Forensische Altersdiagnostik (AGFAD), or Study Group for Forensic Age Diagnosis of the German Society of Forensic Medicine, recommends a general physical examination, a dental anamnesis with visual assessment of the oral cavity (including X-ray imaging where permitted), and a thorough analysis of skeletal development via X-ray of the left hand and wrist.

If the carpal X-ray indicates completion of the maturation process, examination of the clavicles should follow, preferably using conventional radiographic methods or computed tomography [4, 5].

Schmeling et al. [5] published a review article recommending that forensic medical reports adopt the concept of minimum age as the primary criterion for age estimation when interpreting results. Incorporating this approach into the interpretation of medical diagnostic tests provides an additional safeguard of legal certainty for minors, thereby minimizing the risk of ethically unacceptable errors, such as mistakenly classifying minors as adults [6, 7].

Accurately estimating legal age remains a complex and challenging task, often hindered by limited expertise and resources. Many professionals involved in age assessment lack familiarity with standardized methodologies or do not have access to the necessary statistical tools to ensure reliable outcomes. In the specific context of dental age estimation, the challenge is compounded by the limited availability of specialists in forensic odontology, a highly specialized field within dentistry. Forensic odontologists constitute a small professional community, and their presence is not guaranteed in all geographic regions [8], leading to restricted access to expert evaluations in legal contexts [9]. Within these constraints, the development of practical and accessible methods to support the initial assessment of dental structures in age estimation would be especially valuable, particularly in settings where specialized expertise is unavailable.

To address this need, Gambier et al. [10] developed a method based on the assessment of third molar eruption stages, designed to assist forensic consultants in the challenging task of legal age estimation. Prior to this, Olze et al. [11] had evaluated a similar approach, developing a reliable tool that has since been validated in two different populations [12, 13]. However, since tooth eruption is a highly variable process influenced by numerous factors, including diet and certain systemic or local diseases [14, 15], the accuracy of this methodology is inherently dependent on the specific characteristics of each population [16]. Therefore, it is essential to undertake rigorous validation studies, particularly in populations where such research is lacking, such as in many African and Asian cohorts [9, 17].

In the Lebanese population, only one study has been conducted on the eruption of the permanent dentition [18]; however, third molar eruption was not included in the analysis.

In light of the foregoing, the primary objective of this study is twofold. The first is to evaluate the applicability of Gambier’s method [10] for legal age estimation in a sample of Lebanese subadults and the second is to evaluate the specific stages and phases defined by this method in assessing whether an individual has reached the age of 18 years.

## Materials and methods

### Sample

A retrospective sample of 537 healthy Lebanese subjects (239 females and 298 males), aged between 15 and 24 years, was collected (see Table 1). All orthopantomograms (OPGs) were obtained from private orthodontic clinics in Beirut, Lebanon, where they were originally taken for orthodontic treatment as part of pre-treatment planning. Subjects were anonymized, with their sex, date of birth, and date of X-ray acquisition recorded for each OPG.

**Table 1:** Age-and-sex-distribution of the total sample

Age group (years)	Lebanese sample	
	Males	Females
15–15.99	34	33
16–16.99	43	34
17–17.99	44	30
18–18.99	27	18
19–19.99	42	30
20–20.99	31	20
21–21.99	31	31
22–22.99	23	21
23–23.99	23	22
Total	298	239

Inclusion criteria required high-quality OPGs displaying intact third molars. Exclusion criteria included OPGs showing tumors, surgical materials, mandibular or maxillary fractures, gross pathology, prior orthodontic treatment, or signs of infection in the third molar regions. Additionally, OPGs of suboptimal quality that impeded accurate interpretation, as well as radiographs affected by distortion, were excluded. Individuals with a history of third molar extraction, retained primary molars, or agenesis of all four third molars were also omitted from the study. Socioeconomic status and specific ethnic backgrounds were not assessed among participants. Importantly, none of the radiographs were taken specifically for the purposes of this research.

### Data management

All third molars were identified using the two-digit FDI (Fédération Dentaire Internationale) system [19]: 18 for the right maxillary third molar, 28 for the left maxillary third molar, 38 for the left mandibular third molar, and 48 for the right mandibular third molar. Each OPG was anonymized and assigned a unique identification number. Detailed information for each OPG, including identification number, sex, date of birth, date of radiograph acquisition, chronological age, and the stages/phases of each third molar eruption was systematically recorded using Microsoft Excel 2016 for data management. Chronological age was calculated by subtracting the date of birth from the date of the OPG and converting the result into decimal age. This study was conducted in accordance with the ethical standards established by the Declaration of Helsinki (Finland) and its subsequent amendments [20].

## Methodology

### Gambier et al.’s staging system

**Fig. 1:**
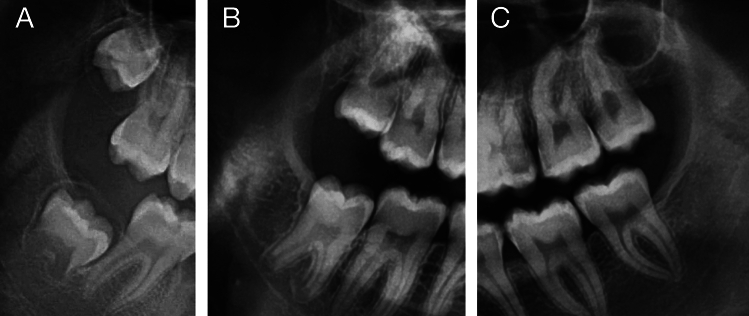
**A** Stage 1 of teeth FDI 18 and 48. **B** Stage 2 of teeth FDI 18 and 48. **C** Stage 3 of teeth FDI 28 and 38

The Gambier et al.’s method [10] utilizes a three-stage scoring system. In stage 1, the third molar has an intact follicle, is positioned under the alveolar bone, and has not yet erupted (Fig. 1A). Stage 2 is characterized by a disrupted follicle and initial eruption, with one or more cusps breaking through the alveolar bone (Fig. 1B). Stage 3 indicates full eruption of the third molar, extending to the occlusal plane (Fig. 1C). For cases where OPGs depicted four assessable third molars, Gambier et al. [9] defined four phases as follows:

- Phase A: All four third molars classified as stage 1.
- Phase B: At least one third molar classified as stage 2.
- Phase C: At least one third molar classified as stage 3.
- Phase D: All four third molars classified as stage 3.

### Image analysis and intra- and inter-observer reliability assessment

All OPGs were examined by a forensic odontologist with 10 years of experience in dental age estimation. Each OPG was assigned a unique identification number to enable randomized and blinded evaluation. To evaluate inter-observer reliability, a second examiner, a dental practitioner with < 1 year of experience in dental age estimation, independently assessed a subset of the OPGs. Additionally, intra-observer reliability was assessed by having the primary examiner re-evaluate a randomly selected subset of 100 OPGs after a 2-month interval. Third molars that were not normally aligned were recorded by the examiners as impacted. Tooth impaction was defined as a deviation from the normal eruption trajectory within the dental arch. The data reported in this study are based on the initial evaluations performed by the primary examiner.

### Statistical analysis

Data processing and analysis were conducted using IBM SPSS Statistics version 29.0 (SPSS Inc., Chicago, IL, USA). Agreement between examiners was evaluated by calculating Cohen’s kappa coefficients to assess both intra- and inter-examiner reliability. Descriptive statistics were initially computed to summarize the characteristics of each stage and phase of third molar eruption as defined by Gambier et al. [10], including mean, standard deviation (SD), median, interquartile range, and overall age range (minimum to maximum). Comparisons of mean ages between different eruption stages and between sexes were performed using Student’s *t* tests. Associations between age categories (≤ 18 years versus > 18 years) and eruption phases were examined with Chi-square tests. Additionally, subgroup analyses focusing on individuals at the age threshold of 18 years, based on both chronological age and the third molar eruption phases according to Gambier et al. [10], were carried out using Chi-square tests with Bonferroni adjustments to correct for multiple comparisons across age groups and eruption phases. Statistical significance was defined at a *p *value < 0.05.

## Results

The age and sex distribution of the participants is detailed in Table 1. Repeated scoring by the same examiner (intra-observer) revealed a very high agreement of 0.95 (95% CI of 0.89–0.93), while inter-observer agreement was 0.91 (95% CI of 0.86–0.91), respectively.

Table 2 presents data on molars that are available and suitable for analysis using the proposed methodology in the collected sample. Among the third molars assessed, the maxillary molars (FDI 18 and 28) were the most frequently evaluable, with only three cases of impaction reported for each. In comparison, the mandibular third molars (FDI 38 and 48) showed higher frequencies of impaction, with 38 cases for tooth 38 and 35 for tooth 48. Missing third molars were also more common in the mandibular arch, with 32 cases for tooth 38 and 51 for tooth 48, compared to the maxillary region. Despite these differences, the overall proportion of evaluable third molars remained high across all quadrants, providing a strong basis for the analysis of eruption stages and phases.

**Table 2:** Proportion of evaluable and non-evaluable third molars in the studied sample

	Number of teeth
Tooth 18 Maxillary right third molar	
Evaluable	483
Missing	51
Impacted	03
Total	537
Tooth 28 Maxillary left third molar	
Evaluable	484
Missing	50
Impacted	03
Total	537
Tooth 38 Mandibular left third molar	
Evaluable	467
Missing	32
Impacted	38
Total	537
Tooth 48 Mandibular right third molar	
Evaluable	451
Missing	51
Impacted	35
Total	537

Table 3 presents the descriptive statistics of chronological age according to the Gambier et al. [10] eruption stages for each third molar, stratified by sex. Mean chronological age increased consistently with each eruption stage, from stage 1 to stage 3, across all four third molars in both males and females. For tooth 18, the mean age in males progressed from 17.37 years in stage 1–21.01 years in stage 3. In females, mean ages for the same tooth increased from 17.51 years in stage 1–21.11 years in stage 3. This upward trend was similarly observed for the other third molars (teeth 28, 38, and 48), with age advancing through successive stages. For each third molar, it can be observed that the minimum age of appearance of the third stage is < 18 years for both sexes.

**Table 3:** Descriptive statistics of the chronological age according to sex and Gambier et al. eruption stages of all third molars (FDI notation)

	Stage	Lebanese sample							
		Males				Females			
		*n*	Mean	Min	Max	*n*	Mean	Min	Max
Tooth 18	1	122	17.37	15.02	23.30	106	17.51	15.01	23.29
	2	77	19.16	15.05	23.57	53	19.54	15.11	23.41
	3	72	21.01	15.14	23.21	53	21.11	15.34	23.61
Tooth 28	1	120	17.35	15.02	23.38	100	17.57	15.01	23.29
	2	89	19.46	15.05	23.57	56	19.21	15.09	23.41
	3	64	20.92	16.14	23.52	55	21.14	15.34	23.61
Tooth 38	1	76	16.94	15.02	22.46	61	17.41	15.01	23.27
	2	118	19.18	15.05	23.30	91	19.15	15.02	23.45
	3	65	20.60	15.42	23.57	56	20.48	16.03	23.61
Tooth 48	1	80	17.06	15.02	23.21	59	17.26	15.01	23.29
	2	115	19.13	15.05	23.30	99	19.34	15.02	23.45
	3	54	20.50	15.42	23.52	44	20.58	16.18	23.61

Descriptive statistics for the Gambier et al. [10] eruption phases, from A to D, are summarized in the Table 4. Mean chronological age increased progressively with each phase for both males and females, confirming a clear association between eruption phase and age. In phase A, the mean age was 16.73 years for males and 16.87 years for females. Phase B was associated with higher mean ages of 18.83 years for males and 18.96 years for females. In phase C, mean ages increased further to 19.87 years in males and 20.02 years in females. Phase D, which reflects complete emergence in the occlusal plane, corresponded to the highest mean ages of 21.18 years in males and 21.01 years in females. In relation to this phase, it can be observed that the minimum age of complete eruption of the four molars is < 18 years.

**Table 4:** Descriptive statistics of the chronological age according to sex and Gambier et al. eruption stages of all third molars (FDI notation)

Phase	Lebanese sample							
	Males				Females			
	*n*	Mean	Min	Max	*n*	Mean	Min	Max
A	57	16.73	15.02	21.23	37	16.87	15.01	23.27
B	101	18.83	15.05	23.30	90	18.96	15.02	23.41
C	35	19.87	15.42	23.38	28	20.02	16.03	23.45
D	27	21.18	17.19	23.52	22	21.01	16.18	23.61

Table 5 presents the distribution of the sample across age categories and Gambier stages (Stages 1–3) for each of the four third molars (teeth FDI 18, 28, 38, and 48), divided by sex. This table shows that 287 males and 202 females with stage 2 are aged 18 and over. In contrast, 228 men and 187 women with stage 3 are 18 years or older.

**Table 5:** Distribution of the sample according to the age categories for each Gambier stage studied for all four third molars (FDI notation)

Age groups	Tooth 18			Tooth 28			Tooth 38			Tooth 48		
	Stage 1	Stage 2	Stage 3	Stage 1	Stage 2	Stage 3	Stage 1	Stage 2	Stage 3	Stage 1	Stage 2	Stage 3
Males												
15–15.99	24	5	0	26	2	0	24	6	1	24	5	1
16–16.99	37	3	1	36	3	1	23	12	1	24	12	0
17–17.99	25	14	3	25	15	3	14	16	7	15	19	7
18–18.99	13	10	3	10	11	5	4	15	4	4	13	5
19–19.99	6	17	15	4	23	11	1	28	10	3	26	8
20–20.99	8	9	10	10	11	8	5	13	10	4	11	7
21–21.99	4	13	10	4	15	10	3	11	10	2	15	8
22–22.99	3	3	14	2	5	13	2	9	9	3	5	9
23–23.99	2	3	16	3	4	13	0	8	13	1	9	9
Females												
15–15.99	27	1	1	26	1	1	17	9	0	20	6	0
16–16.99	25	5	1	22	9	2	19	10	4	15	13	3
17–17.99	18	8	1	19	6	2	5	16	3	8	13	3
18–18.99	7	6	2	6	5	2	3	7	6	1	11	4
19–19.99	12	10	6	10	14	4	7	14	6	6	13	6
20–20.99	5	7	8	4	7	7	1	8	9	2	12	2
21–21.99	7	6	13	7	7	13	7	10	9	4	12	10
22–22.99	2	6	10	1	5	12	1	6	12	1	7	11
23–23.99	3	4	11	5	2	12	1	11	7	2	12	5

Table 6 summarizes the sample age distribution according to sex and the four phases of the Gambier method (Phases A–D). Notably, a high number of subjects over 18 years of age are observed in both males and females in phase B. At the same time, a false positive is observed in both males and females under the age of 18.

**Table 6:** Distribution of the sample according to the age categories for each Gambier phase studied for all four third molars (FDI notation)

Age groups	Males				Females			
	Phase A	Phase B	Phase C	Phase D	Phase A	Phase B	Phase C	Phase D
15–15.99	17	7	1	0	14	8	0	0
16–16.99	21	13	1	0	11	13	4	1
17–17.99	9	18	7	1	4	15	4	0
18–18.99	4	12	2	3	0	9	1	2
19–19.99	3	17	8	4	5	12	3	3
20–20.99	1	12	4	3	1	7	4	2
21–21.99	2	12	2	4	1	13	3	5
22–22.99	0	7	3	5	0	6	4	5
23–23.99	0	3	7	7	1	7	5	4

Table 7 presents the relationship between age classification (under or over 18 years) and third molar eruption phases in both sexes. The analysis demonstrated a good association between advanced eruption phases and the likelihood of being 18 years or older. In Phase A, 21.4% of males and 16.4% of females were under the age of 18. Conversely, Phase D showed that only the 11.9% of males and females in this phase is above the legal age threshold. It is important to mention that only 1 individual, in both the male and female groups, although in phase D, was under 18 years of age.

**Table 7:** Phase distribution according to age (under or over 18 years) in both sexes

Age classification	Males					Females				
	Phase A	Phase B	Phase C	Phase D	Total	Phase A	Phase B	Phase C	Phase D	Total
Lebanese sample										
< 18 years	47	38	9	1	95	29	36	8	1	74
≥ 18 years	10	63	26	26	125	8	54	20	21	103
Total	57	101	35	27	220	37	90	28	22	177

**Fig. 2:**
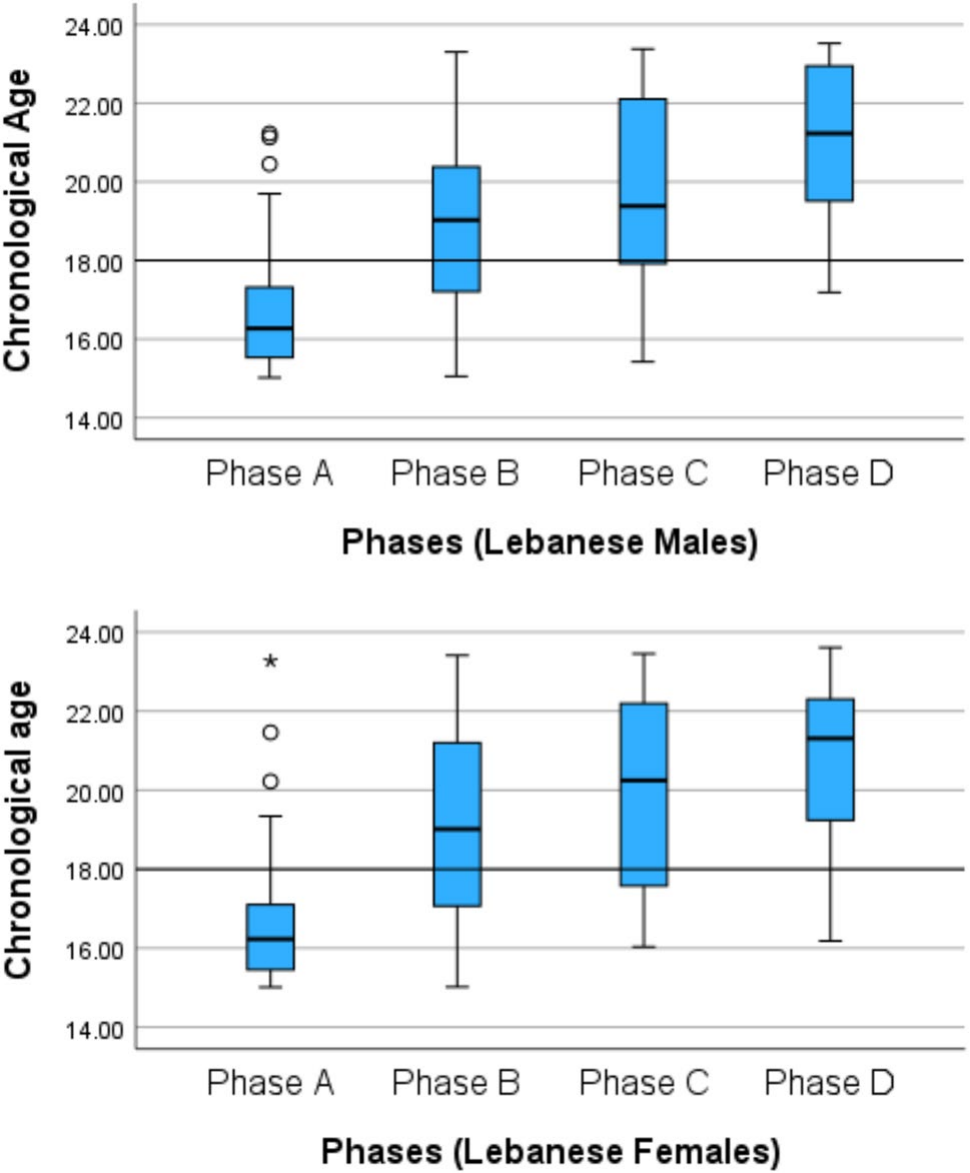
Box-plot of age distribution for each third molar for males and females

Figure 2 illustrates the age distribution corresponding to each third molar eruption stage in the Lebanese sample, according to sex. A clear pattern of age progression was observed across stages, with median and mean ages increasing consistently from stage 1 to stage 3. While males and females demonstrated broadly similar distributions within each stage, females tended to exhibit slightly higher mean ages, particularly in the later stages of eruption. The boxplots also reveal a wide chronological age range within individual stages, with notable overlap between adjacent stages.

## Discussion

This study is the first to investigate third molar eruption as a method for legal age estimation in a Lebanese population. To date, only one prior study has investigated the eruption of permanent teeth in this population [18], and another has explored the effects of impaction on third molar development [21]. Moukarzel et al. [22] evaluated the third molar maturity index (I3M) in Lebanese individuals aged 14–23 years. However, none of these studies focus on a detailed analysis of the eruption stages of all four third molars for the purpose of estimating the age of majority. The present study thus addresses a significant gap in the literature by providing the first comprehensive analysis of all four third molars in a Lebanese cohort, with implications for forensic age assessment.

Owing to ongoing political instability in the Middle East and North Africa (MENA) region, Lebanon has become a key host country for Syrian and Palestinian refugees displaced by conflict [23]. In recent years, the country has also faced severe economic challenges, together with persistent internal and regional pressures, which have heightened dependence on humanitarian aid among both Lebanese citizens and refugee populations [24]. Moreover, according to a baseline survey conducted by UNICEF in 2015–2016 [25], 6% of Lebanese girls and women aged 20–24 were married before the age of 18. To underscore the gravity of the situation, a report by the Mixed Migration Platform [26] highlighted that Lebanon lacks a clear, established framework for age assessment, with no evidence indicating its implementation within the national legal system. The primary aim of this study is to provide forensic experts and other professionals involved in assessing alleged minor migrants with a complementary scientific method to support accurate and statistically robust evaluations.

In the present study, 26% of the total sample (140 subjects) could not be assessed using the Gambier method [10] due to third molar impaction or absence. This proportion falls within the ranges reported in previous research. For example, a meta-analysis of third molar impaction found that approximately 36.9% of subjects globally have at least one impacted third molar [27]. Also, Saputri et al. [28] reported that missing or impacted third molars significantly affect assessments of development stages, since development and eruption can be delayed or altered in impacted teeth. These findings underscore that any method relying on third molar eruption will inevitably reduce sample size when agenesis or impaction is present. Because of this, in the present study, the phase‐based assessments (Phases A–D) had smaller numbers of assessable cases than the stage‐based assessments. This reduction in sample size in some phases may limit statistical power and reduce precision in those phases; however, for the subset of subjects with all four assessable third molars, our findings still follow the eruption patterns, i.e., correlation between the phase and chronological age in late adolescence.

Institutions such as the European Union Agency for Asylum (EUAA) [29], which replaced the European Asylum Support Office (EASO), and scientific bodies like the AGFAD have already developed protocols containing detailed and rigorous guidelines for addressing cases involving uncertainty about the age of undocumented migrant minors [4]. Notwithstanding, since its initial publication, the method proposed by Gambier [10] has demonstrated a relative degree of reliability when applied during the early or preliminary stages of the identification process [9, 30, 31]. In many instances, the personnel involved in the initial stages of foster care are often police officers or paramedical staff who may lack the specialized expertise required to accurately assess newly arrived children in the host country [32]. This method may therefore be recommended as a useful tool, as its straightforward application offers a clear advantage for personnel undergoing training. Assessing the stages of eruption on the OPGs is generally more straightforward than techniques based on tooth mineralization or the degree of root apex closure [33]. However, given the high variability of individual stages in both dental mineralization and eruption [34], relying solely on dental assessment for estimating age in alleged minor migrants is not recommended.

Regarding intra- and inter-observer agreement, the findings indicated close to excellent agreement for both measurements. Cohen’s kappa coefficients measure the extent to which measurements can be replicated (reliability). In this study, values between 0.8 and 0.9 indicated good reliability, while values > 0.90 indicated excellent reliability.

This study has demonstrated that a substantial proportion of subjects classified as stage 2 and phases B and C according to Gambier are 18 years of age or older (Tables 3 and 4). Similar trends have been observed in more geographically distant populations, as reported by Chinni et al. [31] and Švábová Nee Uhrová et al. [30]. This implies that, when considering all four molars collectively, there may be a substantial number of false negatives, that is, individuals who, based on the observed degree of eruption, could be incorrectly classified as being under 18 years of age. Previous studies have suggested that individuals of African descent exhibit accelerated tooth eruption [35], whereas Asian populations, in comparison to Europeans, tend to demonstrate delayed eruption [36]. However, the samples used in many of these investigations often did not fully meet the criteria necessary for establishing valid reference populations [36].

Therefore, considerable caution is warranted when analyzing the eruption stages of third molars within this Lebanese population sample, given the potentially high rate of false negatives associated with such evaluations. In forensic practice, such errors are deemed technically unacceptable [6]. An inaccurate age estimation of this nature may result in more lenient legal treatment typically reserved for minors, and it can also impose additional social costs due to the implementation of special protective measures.

Conversely, only two false positives have been identified, one in each sex, when assessing phase D of the method. This indicates that, when applied to this population sample, the likelihood that an individual classified in the final phase described by Gambier et al. is actually a minor is very low [10]. In this context, such an error would be ethically unacceptable, given its potentially severe legal consequences for a minor mistakenly assessed as an adult based on third molar eruption stages [6].

In cases of uncertainty regarding the age of an alleged minor, particularly when significant doubt persists, age assessments should ideally be conducted by accredited forensic institutes operating under appropriate legal mandates and in accordance with established guidelines, such as those issued by AGFAD, last revised in 2008 [4, 5]. This so-called “three-pillar approach” to age estimation supports a comprehensive diagnostic framework that includes a dental examination comprising a dental radiographic examination and documentation of the dental status. However, discrepancies are observed among the practices of several forensic institutes across Europe that routinely handle legal proceedings involving unaccompanied migrant minors and/or asylum seekers, particularly regarding the inclusion or exclusion of dental anamnesis and dental radiographic exam- ination [37–43]. It was described that in routine forensic practice, dental development is frequently evaluated through direct examination of the oral cavity [37]. This approach may increase the risk of significant errors by the examiner, as it does not allow for assessment of the alveolar eruption of the tooth. In jurisdictions in which the assessment is performed and where it is legally permissible, the ‘three-pillar approach’ is recommended, encompassing the use of an OPG, as this imaging modality affords a more robust and reliable assessment of both the eruption status and developmental stage of the third molars. Nevertheless, it remains essential to comply with the radiation protection principles outlined by the International Commission on Radiological Protection (ICRP), particularly in the case of younger individuals [44].

### Study limitations and future prospects

A primary limitation of this study, which is likely shared by recent investigations assessing the validity of the Gambier et al. method [10], is the high prevalence of impacted third molars (79 cases in the present sample), which clearly hinders the proper application of the proposed methodology. Furthermore, the retrospective nature of this study, relying on a sample of OPGs, limits the ability to directly assess the eruption stage of each third molar within the subject’s oral cavity. Despite a high level of inter-observer agreement, the quality of radiographic images and the superimposition of bone structures [45], particularly in the maxillary region, occasionally result in significant errors in eruption assessment. It is important to emphasize that, in forensic practice, radiograph quality is not always optimal, yet this method demands a high degree of image clarity and definition for accurate evaluation.

Looking ahead, and considering the significant advancements in artificial intelligence (AI) models over the past decade within the field of dental age estimation [46], alongside the inherent subjectivity of traditional techniques [47], it would be valuable to compile large, well-balanced, and unbiased samples to better predict patterns of third molar development and eruption. Notably, Vranckx et al. [48] have developed an AI-driven tool enabling rapid, accurate, and consistent automated measurement of molar angulations on the OPGs. Given the high variability of third molars in both mineralization and eruption [49], AI has the potential to reduce this variability, thereby enhancing the reliability of assessments and providing greater confidence in expert decision-making [50]. Consequently, integrating accurate AI tools to support forensic practitioners is expected to significantly improve diagnostic precision and accuracy.

## Conclusions

This study demonstrates that the method developed by Gambier et al. [10] may be employed in legal age estimation, but it should be applied with caution and restricted to the preliminary stages of assessing alleged undocumented migrant minors. Notably, the considerable inter-individual variability in third molar eruption introduces substantial uncertainty into biological age assessment.

The reliability of this method appears to be influenced by critical factors, including the geographical origin of the population and the prevalence of impacted third molars. In this context, our findings based on a Lebanese sample provide important preliminary data, but their generalizability to other populations may be limited. Consequently, further research involving large, demographically balanced cohorts is warranted, particularly utilizing AI to more precisely investigate developmental and eruption patterns of third molars.

## Data Availability

The data supporting this study’s findings are available on request from the corresponding author.

## Declarations

The study was carried out following the ethical standards of the Declaration of Helsinki (Finland).
